# Influence of cement-augmented pedicle screw instrumentation in an osteoporotic lumbosacral spine over the adjacent segments: a 3D finite element study

**DOI:** 10.1186/s13018-020-01650-5

**Published:** 2020-04-07

**Authors:** Quan-kun Zhou, Fan-hui Zeng, Jian-long Tu, Zhang-qing Dong, Zhi-Hui Ding

**Affiliations:** Nanchang Hongdu Hospital of Traditional Chinese Medicine, 264 Minde Road, Donghu District, Nanchang, 330006 Jiangxi People’s Republic of China

**Keywords:** Cement-augmented pedicle screw;, Osteoporotic lumbosacral spine;, Adjacent segment;, Finite element study

## Abstract

**Abstract:**

**Purpose:**

To compare the effect of conventional pedicle screw (CPS) and cement-augmented pedicle screw instrumentation (CAPSI) on adjacent segment degeneration (ASD).

**Methods:**

A normal male volunteer without a history of spinal disease was selected, lumbar CT data was collected, an intact L3-S1 three-dimensional finite element model was created by software including Mimics, Geomagic, and SolidWorks, and the fixation methods were performed accordingly. A common pedicle screw model and a cement-augmented pedicle screw model of L4–L5 with fusion and internal fixation were constructed. With ANSYS Workbench 17.0, a 500 N load was applied to the upper surface of L3 to simulate the weight of a human body, and a 7.5 N m moment was applied at the neutral point to simulate flexion, extension, left/right bending, left/right rotation of the spine. The peak von Mises stress of intervertebral disc and the range of motion (ROM) on the adjacent segments (L3–4 and L5–S1) were compared.

**Results:**

The validity of the intact model shows that the ROM of the model is similar to that of a cadaveric study. Compared with the intact model, CPS model and CAPSI model in all motion patterns increased the ROM of adjacent segments. The intervertebral disc stress and the ROM of adjacent segments were found to be higher in the CAPSI model than in the CPS model, especially in L3–4.

**Conclusion:**

In general, the biomechanical analysis of an osteoporotic lumbar spine showed that both CPS and CAPSI can increase the ROM and disc stresses of osteoporotic lumbar models, and compared with CPS, CAPSI is more likely to increase the potential risk of adjacent segment degeneration.

## Introduction

During posterior lumbar fusion, it is often necessary to partially or completely remove the facet joint, which causes a loss of stability in the surgical segment. For example, transforaminal lumbar interbody fusion (TLIF) is an effective treatment for degenerative spinal pathologies and has been widely used because of a variety of indications and a high interbody fusion rate. But in the procedure of TLIF, the facet joint need to be remove on one side or double side, which may lead to unstable vertebral body. Therefore, an internal fixation device is often used to maintain stability in the lumbar segment [1, 2]. However, in patients with osteoporosis, conventional pedicle screws (CPS) have the disadvantage of insufficient holding power and bone cement needs to be used to strengthen the pedicle screw fixation to reduce the risk of screw loosening and pseudoarthrosis [3–5]. Cement-augmented pedicle screw instrumentation (CAPSI) has been proved to strengthen the mechanical force on the screw-bone interface so that to reduce implant failure rate by both vivo and vitro studies [3–5]. According to the current clinical investigation and biomechanical studies, CAPSI showed a significantly lower loosening rate (0–4.3%) and higher fusion rate (94.1–100%) compared to regular pedicle screws [4, 6–8].

Although strong internal fixation may lead to decreased spinal mobility in the surgical segment, it also increased intervertebral disc and articular stress in adjacent segments and may increase the potential risk of adjacent segment degeneration (ASD) [9]. ASD refers to the degeneration of cranial and caudal segments after spinal fusion surgery, and the incidence of ASD is approximately 8–100% [10, 11]. Patients may have changes detected only by imaging or clinical symptoms, and severe cases even require surgical revision [12]. At present, the cause of ASD remains unclear. Some scholars believe that internal fixation will accelerate the degeneration of adjacent segments, which is an independent risk factor for ASD [13], while some studies believe that the occurrence of ASD is mainly related to the age of patients and the type of surgery they undergo. There are many factors related to the degeneration of the intervertebral disc in the anterior adjacent segment, and internal fixation is not the main cause of ASD [14]. Therefore, there is no clear conclusion on the degree of influence of ASD on lumbar internal fixation. To date, no biomechanical studies comparing the effects of CPS and CAPSI on ASD have been conducted. Therefore, the authors established an L4–L5 fixed CPS and CAPSI model by the three-dimensional finite element analysis (FEA) method and compared the two internal fixation methods. The influence of adjacent segments provides a reference for the development of surgical strategies in clinical practice.

## Materials and methods

### Construction of a lumbar finite element model

An adult male volunteer was selected and had no history of lumbar disease. CT scan data on the lumbar vertebrae (Siemens, German) were provided by the Department of Radiology at Nanchang Hongdu Hospital of Traditional Chinese Medicine. The scanning parameters were as follows: 155 mAs, 120 KV, and layer thickness of 0.625 mm. The scanning range was from the waist to the tibia. The tomography images were stored in Digital Imaging and Communications in the Medicine format.

**Fig. 1:**
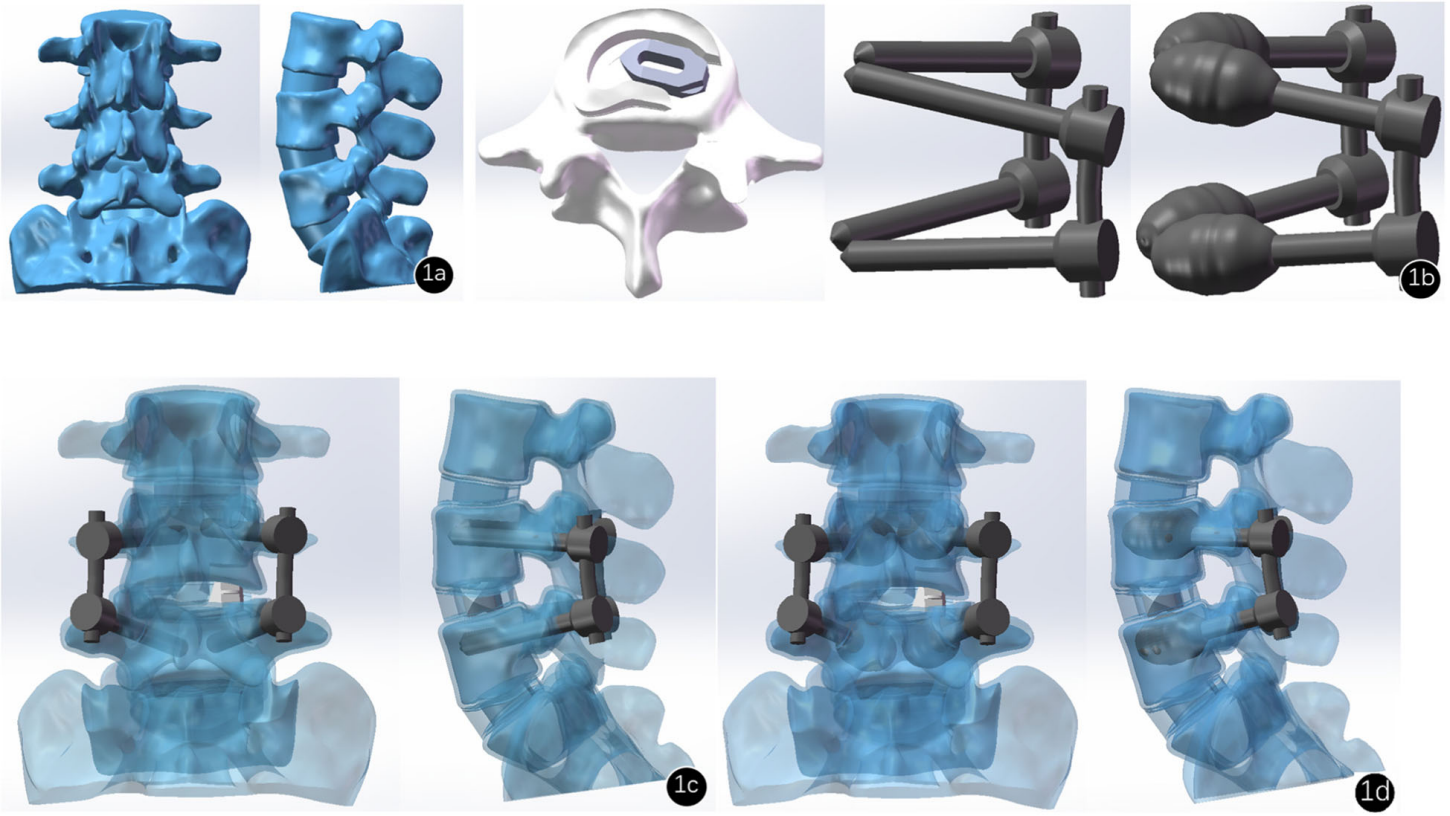
Different types of models. **a** The intact lumbar model. **b** The model of cage in the transforaminal lumbar interbody fusion, the model of conventional pedicle screws, and the model of cement-augmented pedicle screw instrument. **c** The conventional pedicle screw instrumentation model. **d** The cement-augmented pedicle screw instrumentation model

The collected raw data in the DICOM format were imported into Mimics research 19.0 (Materialize, Leuven, Belgium) for three-dimensional reconstruction. Subsequently, the 3D model generated by Mimics was imported into Geomagic Studio 2013 (3D Systems Corporation, South Carolina, USA), and the spikes and the features were deleted, smoothing was performed with a polygon mesh, and the triangles were made more uniform in size. Then, a patch was generated with the following tools: Construct Patches and Grid and Fit Surfaces. The smoothed model was saved and imported into SolidWorks 2017CAD (SolidWorks Corporation, Concord, MA, USA), and cancellous bone, cortical bone, annulus fibrosus, nucleus pulposus, endplate, and articular cartilage models were created in the Parts Interface window; the nucleus pulposus accounted for approximately 50% of the disc area, and the cortical bone and endplate thickness were 0.5 mm and 1 mm [15, 16]. The above parts were integrated into an intact finite element model of lumbar spine (Fig. 1).

### Finite element models of the lumbar fixation condition

Based on the forms of real pedicle screws, cages and bone cement, the models of pedicle screw, cage and bone cement were constructed in the Parts Interface window. The pedicle screw was 45 mm × 6.5 mm (length × diameter), the size of the cage was 12 × 24 mm (height × width), the bone cement was agglomerated, and the volume is approximately 2.5 cm^3^. Subsequently, transforaminal lumbar interbody fusion (TLIF) was performed to remove the right facet joint, cartilaginous endplate, nucleus, and part of the annulus fibrosus of L4–L5. The screws, cages, and bone cement were assembled with the lumbar spine model to construct the CPS and CAPSI models (Fig. 1).

### Material properties and biomechanical evaluation

The mesh model generated in SolidWorks 2017CAD was imported into ANSYS Workbench 17.0 (ANSYS, Ltd., Canonsburg, PA, USA), and previous literature was referenced to set the cortical bone (osteoporosis), cancellous bone (osteoporosis), articular cartilage, endplates, annulus fibrosus, nucleus pulposus, bone cement, cages, and internal fixation (Table 1). The ligaments were simulated using spring elements that were only stressed by pulling force (one ligament stimulated by one spring) [17–20]. The contact type between the models was defined in the connection, where in the facet joint contact type was frictional and the frictional coefficient was 0.2 [21]; the remaining contact types were set to be the bonded mode [20, 22]. Finally, the boundary and loading conditions of the two internal fixation models were set [20, 23]: the inferior surface of the S1 vertebral body was not allowed to move in any direction, a uniformly distributed 500 N surface load was applied on the upper surface of the L3 vertebral body, the pressure direction was vertically downward to simulate a normal body upper body weight, and 7.5 N m of moment was simultaneously applied on the upper surface of the L3 vertebral body in different directions: flexion, extension, left flexion, right flexion, left rotation, and right rotation (six motion states).

**Table 1:** Material properties used in the finite-element model

Component/materials	Young’s modulus E (MPa)	Poisson’s ratio
Cortical bone (osteoporosis)	8040(67% of normal)	0.3
Cancellous bone (osteoporosis)	34(34% of normal)	0.2
Posterior element	3500	0.25
Cartilage	50	0.3
Endplate	1000	0.3
Annulus fibrosus	4.2	0.45
Nucleus pulposus	1	0.499
Ligament		
Anterior longitudinal	20	0.3
Posterior longitudinal	20	0.3
Transverse	59	0.3
Ligamentum flavum	19.5	0.3
Interspinous	12	0.3
Supraspinous	15	0.3
Spinal instrumentation (titanium alloy)	110000	0.3
Spinal cage	3600	0.3
Bone cement	3000	0.4

## Results

### Validation of the model

**Fig. 2:**
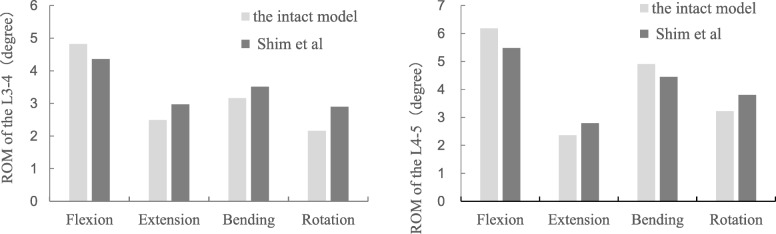
Comparison of the range of motion (ROM) between the intact model and the in vitro study at the L3–4 (left) and L4–5 (right) levels

We assessed the range-of-motion (ROM) of the intact model under different physiological motions, including flexion, extension, lateral bending and rotation with 500N of vertical axial preload and 7.5 N m of moment, which were similar to the cadaveric study. The ROM of L3–L4 and L4–L5 in our results were in good agreement with those in the cadaveric study conducted by Shim et al. [24] (Fig. 2).

### Stress on the intervertebral disc and range of motion

**Fig. 3:**
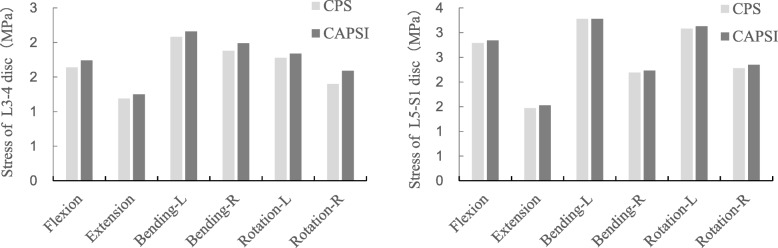
The stresses on the intervertebral disc at L3–4 and L5–S1 in the two fixed models

**Fig. 4:**
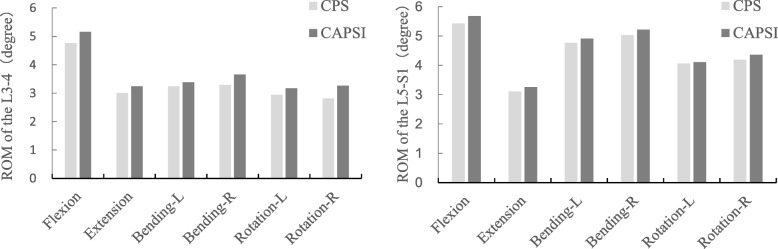
The ROM at L3–4 and L5–S1 in the two fixed models

Compared with the intact model, the CPS and CAPSI models showed increased ROM in the adjacent segments for all motion patterns. The stresses on the intervertebral disc in the CPS model during flexion, extension, left bending, right bending, left rotation and right rotation were 1.64 MPa, 1.19 MPa, 2.08 MPa, 1.88 MPa, 1.78 MPa, and 1.40 MPa, respectively, in L3–L4, and 2.79 MPa, 1.47 MPa, 3.28 MPa, 2.19 MPa, 3.08 MPa, and 2.27 MPa, respectively, in L5–S1 (Fig. 3). The adjacent segmental ROM in the CPS model during flexion, extension, left bending, right bending, left rotation, and right rotation were 4.76°, 3.01°, 3.24°, 3.29°, 2.94°, and 2.81°, respectively, in L3–L4, and 5.43°, 3.11°, 4.77°, 5.03°, 4.06°, and 4.19°, respectively, in L5–S1 (Fig. 4).

**Fig. 5:**
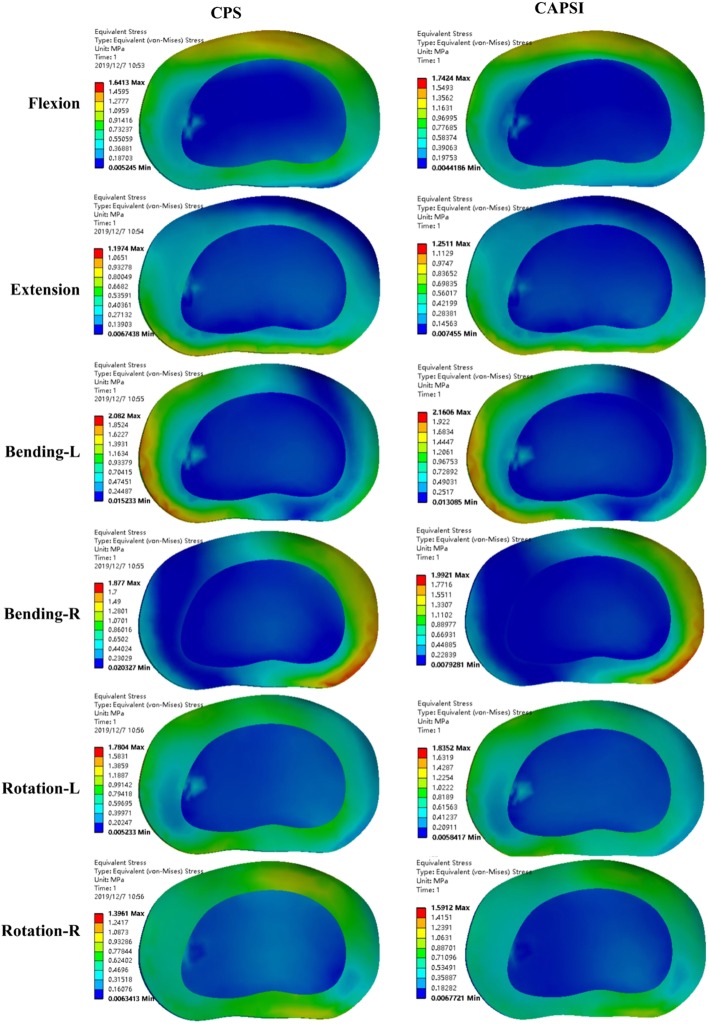
The distribution of the peak von Mises stresses at L3–4

**Fig. 6:**
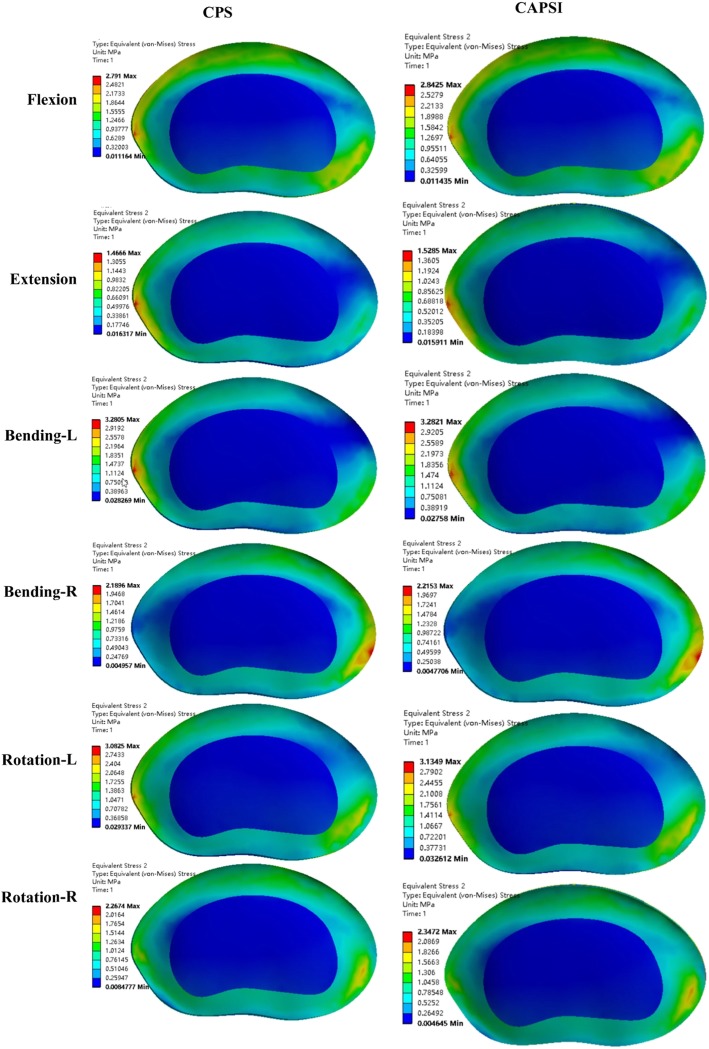
The distribution of the peak von Mises stresses at L5–S1

The stresses on the intervertebral disc in the CAPSI model during flexion, extension, left bending, right bending, left rotation and right rotation were 1.74 MPa, 1.25 MPa, 2.16 MPa, 1.99 MPa, 1.84 MPa, and 1.59 MPa, respectively, in L3–L4, and 2.84 M a, 1.53 MPa, 3.28 MPa, 2.22 MPa, 3.13 MPa, and 2.35 MPa, respectively, in L5–S1 (Fig. 3). The adjacent segmental ROM of the CAPSI model during flexion, extension, left bending, right bending, left rotation, and right rotation were 5.16°, 3.24°, 3.38°, 3.66°, 3.17°, and 3.26°, respectively, in L3–L4, and 5.68°, 3.26°, 4.91°, 5.22°, 4.11°, and 4.36°, respectively, in L5–S1 (Fig. 4). The intervertebral disc stress and the ROM of adjacent segments were found to be higher in the CAPSI model than in the CPS model, especially in L3–4. All the peak von Mises stresses were found at the edge of the fibrous ring, and the stress distributions of the two models were similar (Figs. 5 and 6).

## Discussion

As degeneration of the spine has become more common and more lumbar fusion surgeries have been performed, the number of patients with spinal fixation has gradually increased. Epidemiological surveys show that the number of patients who underwent intraspinal fixation increased by nearly 276.03% from 2004 to 2015 [25]. Therefore, complications related to internal fixation, such as pedicle screw loosening and adjacent segment degeneration, have gradually become more common topics of research. In patients with osteoporosis, the rate of screw loosening in common pedicle screws is approximately 10–62.8% [26, 27]. Biomechanical studies have shown that bone cement strengthening can increase the pull-out force from 147 to 278% [5]. To reduce the occurrence of screw loosening and promote the fusion of the intervertebral space, CAPSI is widely used in patients with various osteoporotic spinal diseases. However, there are still some controversies about the use of CAPSI. For example, will CAPSI accelerate the degeneration of adjacent segments compared to CPS?

In most patients with internal fixation, ASD only manifests as a change detected by imaging, but 6.4 to 10.7% of patients have clinical symptoms [28], and 0–24.4% of patients need revision surgeries due to degeneration in adjacent segments. Among the patients who underwent revision surgery for ASD [29, 30], the reoperation satisfaction rate was only 54%, which was significantly lower than that of patients who did not have ASD and underwent revision surgery (83%) [31]. Therefore, clarifying the impact of CAPSI on the adjacent segments can provide a better reference for surgeons to prevent ASD and develop surgical strategies. To the best of our knowledge, this is the first finite element study comparing the effects of CPS and CAPSI on the adjacent segments.

Lumbar degenerative diseases such as lumbar spinal stenosis occur mostly in the L4–5 segment, and CAPSI is also commonly used in patients with osteoporosis. Therefore, this study used a model of L4–5 segmental fusion to analyses the effect of two different instruments on the adjacent segments. Besides, cadaver testing has demonstrated that a cement volume between 1.5 and 3.0 ml significantly improves screw stability, whereas a volume beyond 3.0 ml does not increase the purchasing strength linearly but results in an increase of cement leakage [32]. Thus, in the present study, the amount of bone cement was assumed as 2.5 ml for every pedicle trajectory. Regarding the intervertebral disc stress, the peak von Mises stress of the L3–4 and L5–S1 segments was greater in CAPSI than that of CPS in all directions, especially in L3–4. After lumbar fusion, the ROM of the segments adjacent to the CPS and CAPSI increased compared with that in the intact model, and the CAPSI model increased more significantly, suggesting that internal fixation increases the ROM of adjacent segments. The data from this study suggest that compared with CPS, although CAPSI can reduce the loosening of internal fixation, it may increase the ROM and intervertebral disc pressure of adjacent segments and accelerate the occurrence of ASD. In addition, in the two fixed models, the ROM and intervertebral disc stress at L3–4 were greater than those at L5–S1, which may be related to S1 being a fixed vertebra; only one vertebral body can be active in the L5–S1 segment, while in L3–4, there are two vertebral bodies that can move.

In general, the greater the strength of internal fixation, the more likely the adjacent segments are unstable and degenerate [13]. After adopting CAPSI in osteoporotic vertebral body, the fixation effect is obviously better than CPS, and the activity of the surgical segment become smaller, which leads to the compensatory increase of the upper and lower vertebral body movement range of the surgical segments, and results in increased stress on adjacent segmental intervertebral discs as well as facet joints followed by the accelerating degeneration of adjacent segments. Schulitz et al. [33] performed respectively an average of 5.7 years and 4.6 years follow-up in 70 patients with simple fusion and 69 patients with posterior lumbar fusion. The ASD incidence of the two groups is 10% and 23%, indicating that the strength of internal fixation and ASD are positively correlated. In addition, stiffer bone cement may cause stress concentration which is transmitted to adjacent discs and vertebral bodies through endplates and then increases the risk of ASD. Kim et al. [34] found when the polymethylmethacrylate (PMMA) filling volume exceeded 30% of the volume of a vertebral body, the level of stiffness in excess of that of normal bone, which was easy to cause adjacent vertebra fracture.

Although this study is based on the physical models of lumbosacral spine, there are still some shortcomings. First, patients requiring CAPSI mostly have degenerative changes in adjacent discs, and previous biomechanical date has proven that this could influence the biomechanics involving adjacent discs. Second, facet joint degeneration was not taken into account in the current analysis. In addition, this study did not analyze mild to severe different osteoporotic models which may lead to a selective bias. Therefore, further cadaver studies and large sample prospective clinical investigation should be undertaken to reach a more precise conclusion.

### Conclusion

In general, the current biomechanical analysis in an osteoporotic lumbar spine showed that both CPS and CAPSI can increase the ROM and disc stresses of osteoporotic lumbar models under different physiological motions, including flexion, extension, left bending, right bending, left rotation, and right rotation. And larger ROM and disc stresses over adjacent segment were found in CAPSI model, which indicate that CAPSI is more likely to increase the potential risk of adjacent segment degeneration.

## Data Availability

The datasets used and analyzed during the current study are available from the corresponding author on reasonable request.

## Abbreviations

CPS: Conventional pedicle screw
CAPSI: Cement-augmented pedicle screw instrumentation
ASD: Adjacent segment degeneration
ROM: Range of motion
FEA: Finite element analysis
TLIF: Transforaminal lumbar interbody fusion
